# The Role of Mitochondria in Sex-Dependent Differences in Hepatic Steatosis and Oxidative Stress in Response to Cafeteria Diet-Induced Obesity in Mice

**DOI:** 10.3390/nu11071618

**Published:** 2019-07-16

**Authors:** Juliana Morais Mewes, Fabiana Rodrigues Silva Gasparin, Tiago Yoshida, Mariana Amâncio Daniel da Silva, Maria Raquel Marçal Natali, Paulo Francisco Veiga Bizerra, Karina Sayuri Utsunomiya, Eduardo Hideo Gilglioni, Marcio Shigueaki Mito, Gislaine Cristiane Mantovanelli, Byanca Thais Lima de Souza, Eduardo Makiyama Klosowski, Emy Luiza Ishii-Iwamoto, Jorgete Constantin, Rodrigo Polimeni Constantin

**Affiliations:** 1 Department of Biochemistry, Laboratory of Biological Oxidations and Laboratory of Experimental Steatosis, State University of Maringá, Maringá 87020-900, Paraná, Brazil; 2 Department of Morphophysiological Sciences, State University of Maringá, Maringá 87020-900, Paraná, Brazil

**Keywords:** cafeteria diet, oxidative stress, mitochondrial energy metabolism, ER stress

## Abstract

Female mice fed a cafeteria diet (FCaf) develop higher liver steatosis and oxidative stress than males (MCaf) as a consequence of unresolved ER stress. Here, we investigated whether mitochondria play a role in this sex difference. The isolated mitochondria from FCaf showed more signs of oxidative stress than those of MCaf, correlated with a reduced content of GSH, increased amount of reactive oxygen species (ROS), and lower activities of enzymes involved in ROS neutralisation. Mitochondria from FCaf and MCaf livers exhibited lower rates of succinate-driven state III respiration and reduced ATPase activity in intact coupled mitochondria compared to their controls fed a standard diet (FC and MC), with no differences between the sexes. Fatty acid oxidation in mitochondria and peroxisomes was higher in MCaf and FCaf compared to their respective controls. In the intact perfused liver, there was no difference between sex or diet regarding the fatty acid oxidation rate. These results indicated that cafeteria diet did not affect mitochondrial energy metabolism, even in FCaf livers, which have higher steatosis and cellular oxidative stress. Nevertheless, the increase in mitochondrial ROS generation associated with a decrease in the antioxidant defence capacity, probably contributes to inducing or reinforcing the ER stress in FCaf livers.

## 1. Introduction

The westernised lifestyle is often associated with the consumption of diets that offer an overload of nutrients, leading to obesity and several related metabolic disturbances, including non-alcoholic fatty liver disease (NAFLD). A recent investigation in our laboratory [1] revealed sex differences in the development of NAFLD in a model of obesity induced by a cafeteria diet in mice. Female mice have more extensive hepatic steatosis and signs of oxidative stress compared to males, despite both groups presenting similar body weight gains and adiposity indexes. Additionally, livers from obese males exhibit higher hepatic *Fgf21* expression and FGF21 protein abundance and elevated FGF21 serum concentrations. The observation that such changes in FGF21 levels do not occur in obese females, which instead exhibit features of unresolved ER (endoplasmic reticulum) stress, led us to suggest that the hepatic steatosis induced by the cafeteria diet in mice is directly related to ER stress [1]. This assumption is in line with recent evidence that the ER acts as a sensor of metabolic derangement associated with an imbalanced diet, which activates an adaptive UPR (unfolded protein response), thus directing cells for either survival or death [2,3]. In our study, we have collected evidence that the branch of UPR involving *Fgf21* up-regulation, in order to resolve the ER stress, seems to be effective in males but not in females under cafeteria diet-induced nutritional stress, which makes the females susceptible to the development of hepatic steatosis and cellular oxidative stress [1].

The ER interacts with most cellular compartments, including the plasma membrane, late endosomes, peroxisomes and mitochondria [4,5]. Mitochondria are interconnected with the maintenance of ER homeostasis through cellular redox potential and energy metabolism. The mitochondrial generation of ATP is required for chaperone function, protein degradation through autophagy and the maintenance of Ca^2+^ stores and redox homeostasis. Under ER stress conditions, UPR is activated in order to restore proper protein folding and ER homeostasis [6,7]. Under prolonged stress, a chronic activation of UPR signalling can eventually induce programmed cell death in order to eliminate stressed cells [8]. Mitochondria and the ER interact through specialised membrane systems referred to as mitochondrial-associated membranes (MAM), by which the organelles can intermix and exchange cell signals such as reactive oxygen species (ROS) and calcium ions [9,10].

Reactive oxygen species (ROS) produced by mitochondria represent a potent inducer of ER stress, and the involvement of ER stress in the establishment of NAFLD/NASH has already been demonstrated [11]. In our previous study, we found that livers from females fed a cafeteria diet have a high content of lipid peroxidation products, indicating ROS-induced damage, as well as exhibiting higher steatosis and NAS scores compared to the males fed a cafeteria diet [1].

The mechanisms linking ER stress and mitochondrial bioenergetics are poorly characterised. In tunicamycin-treated HeLa cells [12], the inter-organelle calcium transfer between the ER and mitochondria seems to promote mitochondrial respiration during the early phase of ER stress via the calcium-dependent activation of dehydrogenase, thus promoting increased ATP production. However, under prolonged ER stress, a calcium overload from the ER into the mitochondria is suggested as a cause of the decrease in mitochondrial energy metabolism and apoptosis [13]. In another study, the coupling between mitochondrial function and ER stress was studied in sk-Hepl human liver cell culture. The mitochondrial function was disrupted by oligomycin and cellular ER stress was shown to be linked to Ca^2+^ and p38 MAPK activation [14]. It was also reported that the treatment of interleukin 3-dependent Bak^−/−^ Bax^−/−^ haematopoietic cells with tunicamycin leads to the sustained activation of the UPR and to a decrease in energy metabolism, particularly in mitochondrial metabolism, with a decrease in mitochondrial membrane potential, oxygen consumption, and cellular ATP levels [15].

The ER stress and mitochondrial dysfunction observed in isolated cells treated with tunicamycin or oligomycin generally results in extreme UPR-induced cell death. In our previous study [1], we demonstrated liver metabolic dysfunctions related to unresolved ER stress in females but not in males fed a cafeteria diet, i.e., in a more physiologically relevant condition and without the use of drugs. The experimental model of cafeteria diet-induced obesity in females and males therefore emerges as a valuable approach with which to investigate the possible role of mitochondria in an ER stress condition. Therefore, in this study, we conducted a comparative investigation of mitochondrial functions in mitochondria isolated from mice of both sexes fed standard or cafeteria diets. Several parameters related to ROS generation, antioxidant systems and energy metabolism of isolated mitochondria were assessed. To examine the physiological relevance of data obtained in isolated mitochondria, the metabolic fluxes linked to energy metabolism were also evaluated in the intact perfused liver.

## 2. Materials and Methods 

### 2.1. Materials

The liver perfusion apparatus was built in the workshops of the State University of Maringá. Enzymes and coenzymes were purchased from the Sigma Chemical Company (St. Louis, MO, USA) as reagent-grade chemicals, salts, buffers, and substrates. [1-^14^C] octanoic acid (25 mCi/mmol) was purchased from New England Nuclear (Boston, MA, USA). All other reagents were obtained at the highest available grade.

### 2.2. Animals

Weaned, 21-day-old male and female Swiss mice were provided by the Animal Facility of the University of Maringá. Animals were kept in polypropylene cages (3 animals/box) in a light-dark cycle of 12 h at 22–24 °C. Mice were arbitrarily divided into 4 groups: cafeteria males (MCaf); cafeteria females (FCaf), control males (MC) and control females (FC). Control groups were fed *ad libitum* with rodent standard diet, while cafeteria groups were fed with Brazilian industrial food (cafeteria diet) for 14 weeks. The body weight of animals was assessed at the beginning of the study (at Day 1) and at the end (Day 98). In the 14th week, for most of the experiments, the mice were euthanised with an overdose mixture of sodium thiopental-lidocaine (150–4 mg/kg, intraperitoneally) before removal of the liver or adipose tissues. The tissues were removed using scissors and tweezers and immediately rinsed with a sterile sodium chloride solution (0.9%). Some experiments were performed shortly after tissue removal, whereas others involved the tissues being stored at −80 °C until processing. For the surgical procedure of liver perfusion experiments, the animals were anaesthetised by an intraperitoneal injection of sodium thiopental-lidocaine (50–4 mg/kg). In all experimental protocols, the mice were fasted overnight before surgical removal of the liver or adipose tissues. The experiments were conducted in the morning (8:00 to 9:00 a.m.). Animals were handled in accordance with the guidelines of the Animal Experimentation of Ethics Committee of University of Maringá (registered number 7405160816).

### 2.3. Diet

The control groups had free access to water and standard rodent chow (Nuvilab-Nuvital^®^, Colombo, Brazil). This standard rodent chow is composed of whole ground corn, soybean meal, and wheat bran and is supplemented with minerals, amino acids, and vitamins, totalling 16.15 kJ/g (64% carbohydrate, 26% protein, and 10% lipid). The cafeteria groups had free access to Brazilian industrialised and high-density caloric food, such as cheese- or bacon-flavoured chips, marshmallows, peanut candy, filled and wafer cookies, sausage, mortadella, and soda, which were all offered in excess [1,16]. These foods have been selected to mimic the eating behaviors prevalent in Western countries, which are associated with obesity and related metabolic disturbances [17,18]. The energy density of the cafeteria diet (taken from the daily offering of each component) totalled 16.28 kJ/g on average (73% carbohydrate, 10% protein, and 17% lipid). This macronutrients proportion is comparable with other studies using cafeteria diet, which also contain higher amounts of carbohydrate and lipid than protein [18,19,20]. The energy density from carbohydrate, protein, and lipid in control and cafeteria mice was calculated in accordance with the nutritional information provided by the manufacturers. The sources of the main macronutrients were variable (type and proportion) in each food of the cafeteria diet and were provided by the following: corn and wheat flour, textured soy protein, poultry and pork meat, pork skin and giblets (liver, kidneys, heart), poultry fats, hydrogenated vegetable fat, corn and cassava starch, whey powder, gelatine, roasted peanuts, sugar, glucose syrup, invert sugar, and/or corn cream. The cafeteria animals also had free access to the standard diet and water. Both the standard and cafeteria diets were replaced daily with fresh food. Food ingestion (g and kJ) was evaluated by weighing each constituent of the offered diet and corresponding leftovers on the following day.

### 2.4. Adiposity Index

At the end of treatment, gonadal, mesenteric, subcutaneous and retroperitoneal fat deposits were weighed, and the adiposity index was calculated from the sum of these tissue weights, expressed in g per 100 g body weight.

### 2.5. Liver Histochemical Analysis and Determination of the Total Lipid Content

For histochemical analysis, liver samples were rapidly removed, frozen in liquid nitrogen, stored at −80 °C, and cut into 10 µm-thick semi-serial histological sections using the Leica^®^ CM1850 cryostat (Leica Biosystems, Wetzlar, Germany). Sudan III staining was performed according to a standard method (Bancroft and Gamble, 2008) for the histochemical identification of lipid vesicles. Images were captured with a 20× objective (100 images/group, 25 images/animal) of an optical microscope (Olympus BX41^®^, Tokyo, Japan) with a QColor3^®^ camera (Olympus American Inc., Canada), coupled to the software Q-Capture^®^. Centrilobular veins in the centre of the images were used as criteria in the choice of fields to be captured. The Image Pro-Plus^®^ 4.5 software was used to determine the area occupied by lipid inclusions in each image, which is related to the total area of the image, and obtained by calculating the area of lipid inclusions in µm × 100^−1^ × total area of the image^−1^.

### 2.6. Liver Mitochondria Isolation

The livers were removed and immediately cut into small pieces. These fragments were suspended in a medium containing 0.2 M mannitol, 75 mM sucrose, 2.0 mM Tris-HCl (pH 7.4), 0.2 mM ethylene glycol-bis (2-aminoethylether)-*N*,*N*,*N*′,*N*′-tetra-acetic acid (EGTA), 0.1 mM PMSF, and 50 mg% (*w*/*v*) fatty acid free bovine serum albumin. Homogenisation was carried out in the same medium by means of a Dounce homogeniser. After homogenisation, the mitochondria were isolated by differential centrifugation [21] using a sucrose–mannitol isolation medium, and then suspended in the same medium, which was kept at 0–4 °C. The protein content of isolated mitochondria was measured employing Folin and Ciocalteu’s phenol reagent [22] using bovine serum albumin as a standard. Disrupted mitochondria were obtained by repeated freeze-thawing events in liquid nitrogen.

### 2.7. Determination of Oxidative Injury Parameters

Disrupted mitochondria were assayed for oxidative damage parameters. Lipid peroxidation was evaluated by thiobarbituric acid reactive substances (TBARS detection), predominantly malondialdehyde (MDA) [23]. Aliquots of disrupted mitochondria (4 mg protein) were added to 1.7 mL of a solution containing 0.4% SDS (sodium dodecyl sulphate), 7.5% acetic acid and 0.25% TBA (thiobarbituric acid). After 1 h incubation at 95 °C, the MDA-TBA complex was extracted with 1.7 mL n-butanol/pyridine 15:1 (*v*/*v*) and the absorbance was determined at 532 nm (ε = 1.56 × 10^5^ M^−1^ cm^−1^). The amount of lipoperoxides was calculated from the standard curve prepared with 1,1′,3,3′-tetraethoxypropane, and the values were expressed as nmol/mg protein.

The carbonylated protein content was determined with the use of 2,4-dinitrophenylhydrazine (DNPH) [24], following modifications [25,26,27]. Aliquots of disrupted mitochondria were homogenised in a medium containing 50 mM phosphate butter and 1 mM EDTA (pH 7.4), vortexed and centrifuged at 10,000× *g* for 15 min at 4 °C. The supernatant was transferred to tubes with 1% streptomycin sulphate in 50 mM HEPES (9:1 *v*/*v*; pH 7.2), incubated for 15 min at room temperature and centrifuged at 6000× *g* for 10 min. Next, 500 μL of the supernatant was transferred to Eppendorf tubes with DNPH in 2 M HCl (1:1 *v*/*v*) and incubated in the dark for 1 h at room temperature with vortexing every 15 min. Blanks with supernatant and 2 M HCl were performed in parallel. Then, 500 μL of 20% trichloroacetic acid was added and centrifuged at 11,000× *g* for 10 min. The supernatant was discarded, and the pellets were washed with 1.0 mL ethanol-ethyl acetate (1:1, *v*/*v*) before being centrifuged 3 times at 10,000× *g* for 10 min to remove free reagents. The precipitated protein was dissolved in 1.0 mL of 6 M guanidine hydrochloride under vortexing, incubated for 15 min at 37 °C, and centrifuged at 10,000× *g* for 10 min at room temperature. The carbonyl group content was calculated using the molar absorption coefficient for aliphatic hydrazones at 370 nm of 22,000 M^−1^ × cm^−1^ and expressed as nmol/mg protein.

### 2.8. Determination of Redox State Parameters

Disrupted mitochondria were assayed for reduced glutathione (GSH), reactive oxygen species (ROS) and protein reduced thiol contents. The GSH contents were measured fluorometrically using ο-phthalaldehyde (OPT) [28]. The samples were added to a medium containing 0.1 M phosphate buffer and 5.0 mM EDTA (pH 8.0). The reaction was started by adding 100 μL of OPT solution (1 mg/mL, in methanol). The fluorescent product GSH-OPT was measured fluorometrically (350 nm excitation and 420 nm emission) after an incubation period of 15 min at room temperature. The results were expressed as μg GSH/mg protein in the mitochondrial suspension.

Total ROS content was quantified via the 2′,7′-dichlorofluorescein-diacetate (DCFH-DA) assay, as previously described [29]. Acetate groups were removed by esterases, producing the reduced DCFH, which can be oxidised by peroxides to the fluorescent oxidised 2′,7′-dichlorofluorescein (DCF) in the presence of ROS. The formation of DCF was measured immediately after stopping the reaction on ice with a spectrofluorometer in which the excitation and emission wavelengths were set to 504 and 529 nm, respectively. A standard curve with oxidised DCF was used to express the results as nmol/mg protein.

The protein thiol (sulphhydryl) contents were determined using the compound 5,5-dithiobis 2-nitrobenzoic acid (DTNB) [30], with modifications [31]. DTNB reacts with thiol groups to yield a coloured product, providing a reliable method to measure protein thiols in solution. For this purpose, proteins in disrupted mitochondria (1.5 mg protein) were precipitated with 20% trichloroacetic acid. After centrifugation at 10,000× *g* for 3 min, the pellet was suspended in a medium containing 0.1 M NaH_2_PO_4_—5 mM EDTA and 10 mM DTNB. After 15 min in the dark (room temperature), the absorbance against blank was determined at 412 nm. The blank consisted of 0.1 M NaH_2_PO_4_—5 mM EDTA and 10 mM DTNB. The protein thiols content was calculated using a molar extinction coefficient of 1.415 × 10^4^ M^−1^ cm^−1^ and expressed as μmol/mg protein.

### 2.9. Determination of Antioxidant Enzyme Activities

The antioxidant enzymatic activities were assessed in the supernatant of disrupted mitochondria. The catalase (CAT) activity was estimated by measuring the change in absorbance at 240 nm using H_2_O_2_ as a substrate. The enzyme activity was expressed as H_2_O_2_ consumed/min × mg protein, using the molar extinction coefficient of H_2_O_2_ of 33.33 M^−1^ × cm^−1^ [32]. The superoxide dismutase (SOD) activity was estimated by its capacity to inhibit the pyrogallol autoxidation in alkaline medium at 420 nm [33]. The amount sufficient to inhibit the enzyme reaction by 50% (IC_50_) was defined as 1 unit of SOD, and the results were expressed as U/mg protein. Glutathione peroxidase activity assessed in the mitochondrial matrix (GPx1) was determined using H_2_O_2_ as a substrate in the presence of NADPH and GSH, by monitoring the decrease in absorbance due to NADPH oxidation at 340 nm over a period of 90 s [34]. The enzyme activity was expressed as nmol of NADPH oxidised/min × mg protein (ε, 6220 M^−1^ × cm^−1^). Glutathione reductase (GR) activity was determined by monitoring the decrease in absorbance at 340 nm due to the oxidation of NADPH [35]. The decrease in absorbance was measured for 90 s, and the initial rate (15 s) was extrapolated to calculate the enzyme activity by using the molar extinction coefficient of NADPH. The enzyme activity was expressed as nmol of reduced NADPH/min × mg protein. Mitochondrial thioredoxin reductase (TrxR2) activity was measured by the increase in absorbance due to the reduction of 5,5′-dithiobis(2-nitrobenzoic acid) (DTNB) with NADPH to 5-thio-2-nitrobenzoic acid (TNB), which produces a strong yellow colour that is measured at 412 nm (ε_412_ = 1.415 × 10^4^ M^−1^ cm^−1^) [36]. The enzyme activity was expressed as nmol/min × mg protein.

### 2.10. Determination of the Respiratory Activity of Isolated Mitochondria

The oxygen consumption by the isolated liver mitochondria was determined by polarography [37], using a Clark-type electrode (Yellow Springs Instruments, Yellow Springs, OH, USA). For measurements of respiration driven by the oxidation of β-hydroxybutyrate and succinate, intact mitochondria (0.6 to 1.0 mg protein/mL) were incubated in a closed plexiglass chamber maintained under agitation and warmed to 37 °C. Overall, 2.0 mL of reaction medium contained 0.25 M mannitol, 10 mM KCl, 5 mM potassium phosphate monobasic, 10 mM TRIS-HCl (pH 7.4), 0.2 mM EDTA and 50 mg% fatty-acid-free BSA (*w*/*v*). The reaction was initiated by the addition of succinate (10 mM) or β-hydroxybutyrate (10 mM). The rates of oxygen uptake were measured after the addition of 125 μM ADP (state III respiration) and after ADP exhaustion (state IV respiration). The RC and ADP/O ratios were determined [38]. The ADP/O ratio represents the amount of ADP added (in mol) to the incubation system divided by the amount of oxygen consumed (in atom-grams) during the active phase of respiration (state III respiration). The respiratory control ratio (RC) was calculated as the rate of active respiration following ADP addition (state III respiration) divided by the rate of respiration after ADP exhaustion (state IV respiration).

For fatty acid β-oxidation, mitochondria (0.6 to 1.0 mg protein/mL) were incubated in 2.0 mL of a reaction medium containing 2.0 mM potassium phosphate monobasic, 0.1 mM EGTA, 130 mM potassium chloride, 5 mM magnesium chloride, 0.1 mM 2,4-dinitrophenol (DNP), 2.5 mM l-malate, 10 mM HEPES (pH 7.2) and 50 mg% fatty acid-free BSA [39]. The reactions were initiated by the addition of (a) 20 mM octanoyl-CoA + 2.0 mM l-carnitine, (b) 20 mM palmitoyl-CoA + 2.0 mM l-carnitine, or (c) 20 mM palmitoyl-l-carnitine.

### 2.11. ATPase Activity

The mitochondrial ATPase activity was assayed by measuring phosphate release [40]. When intact mitochondria were used as an enzyme source, the reaction medium contained 200 mM sucrose, 10 mM Tris–HCl (pH 7.4), 50 mM KCl, 0.2 mM EGTA, and, when required, 100 μM 2,4-dinitrophenol. For incubation with the disrupted mitochondria, the medium contained 20 mM Tris–HCl (pH 7.4). The reaction was started by the addition of 5 mM ATP, incubated for 20 min at 37 °C and stopped by the addition of ice-cold 5% trichloroacetic acid. Phosphate was measured colorimetrically in a spectrophotometer [41]. When coupled mitochondria were assayed, approximately 1 mg/mL of mitochondrial protein was used as an enzyme source, and when uncoupled or disrupted mitochondria were assayed, approximately 0.5 mg/mL of mitochondrial protein was used.

### 2.12. Peroxisomal Fatty Acyl-CoA Oxidase Activity

The peroxisomal fatty acyl-CoA oxidase activity was measured fluorometrically by the method based on H_2_O_2_-dependent oxidation [42], following modifications [43]. The assay was based on the determination of H_2_O_2_ production in a reaction catalysed by exogenous peroxidase, which was coupled to the oxidation of DCFH-DA into the highly fluorescent compound DCF. The enzyme activity was monitored in real-time by recording the variation in fluorescence. After the addition of the peroxisome-enriched fraction (0.3 mg protein/mL), the reaction was initiated with the addition of the substrate, octanoyl-CoA or palmitoyl-CoA (final concentration of 30 μM). The increase in fluorescence (excitation, 503 nm; emission, 529 nm) was recorded over a period of 10 min, and the activity of fatty acid acyl-CoA oxidase was expressed as pmol DCF produced/min × mg of peroxisomal protein.

### 2.13. Liver Perfusion Experiments

The experiments were performed at the end of treatment (14 weeks). For the surgical procedure, animals (control and cafeteria mice) were anaesthetised with a mixture of sodium thiopental-lidocaine injection (50–4 mg/kg, intraperitoneally). To perform haemoglobin-free, non-recirculating perfusion [44], the livers of overnight (12 h) fasted mice were used in all perfusion experiments. After cannulating the portal and cava veins, the liver was positioned in a plexiglass chamber. A constant flow was maintained by a peristaltic pump (Minipuls 3, Gilson, France) and adjusted to 8–10 mL/min, depending on liver weight. The perfusion fluid was the fatty acid (FA)-free bovine serum albumin Krebs/Henseleit-bicarbonate buffer (pH 7.4) saturated with a mixture of oxygen and carbon dioxide (95:5), using a membrane oxygenator with simultaneous temperature adjustment to 37 °C. The Krebs/Henseleit-bicarbonate buffer composition was as follows: 115 mM NaCl, 25 mM NaHCO_3_, 5.8 mM KCl, 1.2 mM Na_2_SO_4_, 1.18 mM MgCl_2_, 1.2 mM NaH_2_PO_4_, and 2.5 mM CaCl_2_. In this experimental protocol, the octanoate (0.3 mM) + [1-^14^C]octanoate (0.01 μCi/mL) mixture was infused as sodium salt complexed with FA-free bovine serum albumin (50 μM). The use of [1-^14^C]-labelled FAs is effective at measuring the citric acid cycle (CAC) flux via acetyl-CoA labelling [45]. Consequently, ^14^CO_2_ production can be regarded as a CAC activity indicator. At the end of the perfusion experiments, the livers were removed and weighed to allow precise metabolic calculation.

After the stabilisation of oxygen consumption, experiments were initiated, where the effluent fluid samples were collected at 4 min intervals and analysed for their metabolic contents. Acetoacetate and β-hydroxybutyrate were assayed by means of standard enzymatic procedures [46]. The oxygen concentration in the outflowing perfusate was continuously monitored by a Teflon-shielded platinum electrode adequately positioned in a plexiglass chamber at the perfusate output [44,45]. The carbon dioxide produced by [1-^14^C]octanoate was measured by trapping ^14^CO_2_ in phenylethylamine [47]. Radioactivity was measured by liquid scintillation spectroscopy. The following scintillation solution was used: toluene/ethanol (2/1) containing 5 g/L 2,5-diphenyloxazole and 0.15 g/L 2,2-p-phenylenebis(5-phenyloxazole). The metabolic rates were calculated from the input and output difference and the total flow rate, and analysed in reference to the liver wet weight. All metabolic fluxes in control and cafeteria mice livers were expressed as µmol/min × g wet liver.

### 2.14. Analytical Assays of Liver Perfusion Experiments

After the stabilization of oxygen consumption, experiments were initiated, and the effluent fluid samples were collected at 4 min intervals and analyzed for their metabolic contents. Acetoacetate and β-hydroxybutyrate were assayed by means of standard enzymatic procedures [46]. The oxygen concentration in the outflowing perfusate was continuously monitored by a teflon-shielded platinum electrode adequately positioned in a plexiglass chamber at the perfusate output [44,45]. The carbon dioxide production from [1-^14^C]octanoate was measured by trapping ^14^CO_2_ in phenylethylamine. Radioactivity was measured by liquid scintillation spectroscopy. The following scintillation solution was used: toluene/ethanol (2/1) containing 5 g/L 2,5-diphenyloxazole and 0.15 g/L 2,2-p-phenylenebis(5-phenyloxazole). The metabolic rates were calculated from the input and output difference and the total flow rate and analyzed in reference to the liver wet weight. All metabolic fluxes in control and cafeteria mice livers were expressed as µmol/min × g wet liver.

### 2.15. Statistical Analysis

The data in the Figures and Tables are expressed as the mean ± standard error (SE). Significant differences between means were identified by two-way ANOVA with the Newman-Keuls post-hoc test or Student’s *t*-test according to the context. The results are given in the text as probability values (*p*). *p* < 0.05 was adopted as the level of significance. Statistical analysis was performed using the Graphic Pad Prism software version 3.0.

## 3. Results

### 3.1. Biometric Parameters and Liver Histochemical Analysis

**Figure 1:**
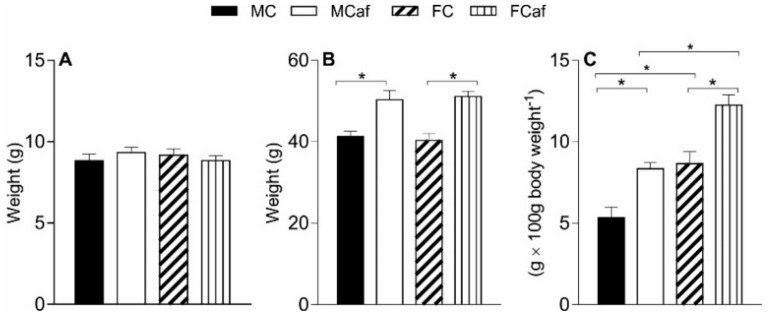
Biometrical parameters of male and female mice fed a standard or a cafeteria diet. Initial body weight (**A**) was determined at the beginning of treatment. Final body weight (**B**) was determined after 14 weeks of treatment. The adiposity index (**C**) was calculated from the sum of the gonadal, mesenteric, subcutaneous and retroperitoneal fat weights after 14 weeks of treatment and expressed in g per 100 g body weight. Each value is mean ± SEM of 6–10 animals per group. * Indicates *p* < 0.05 according to ANOVA. MC—male fed a standard control diet; MCaf—male fed a cafeteria diet; FC—female fed a standard control diet; FCaf—female fed a cafeteria diet.

Biometric parameters of male and female mice fed a standard (MC and FC) or cafeteria diet (MCaf and FCaf) are shown in Figure 1. As previously reported in cafeteria diet-induced obese mice [1], there was no significant difference in the initial weight between the groups (Figure 1A); however, at the end of the experimental period (14th week), a significant increase in body weight was found in the cafeteria diet-fed groups, of 21.7% for MCaf and 26.7% for FCaf, compared to their respective counterparts (MC and FC), with no effect of sex between the groups (Figure 1B). The amount of body fat was also measured in the groups as the adiposity index. Effects of both diet and sex were found. The cafeteria diet induced a significant increase in the percentage of body fat (Figure 1C) in both sexes, and the relative increase was higher in males (+65.4%) and females (+41.6%) compared to MC and FC, respectively. Females fed a standard diet (FC) had a higher absolute adiposity index than males (MC; +71.7%). The cafeteria diet increased the adiposity index in FCaf by 41.6% and in MCaf by 65.4% when compared with their counterparts fed standard diet. 

**Figure 2:**
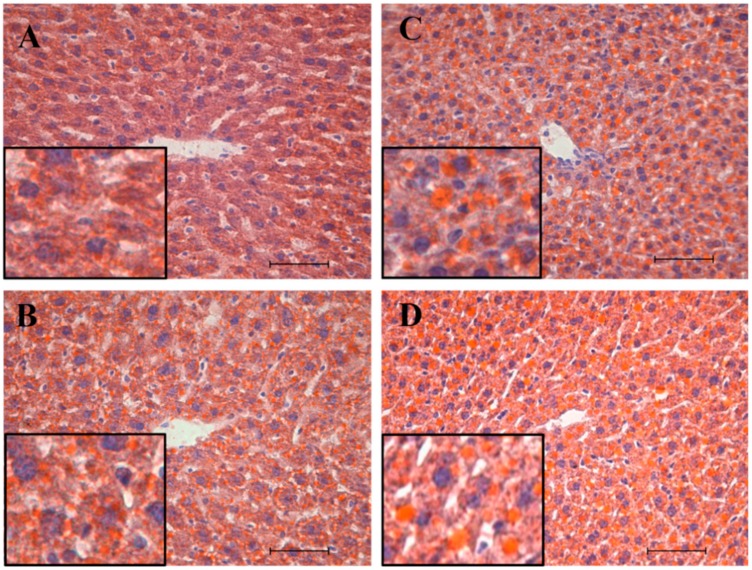
Representative images of 20× of Sudan III staining of the liver of male and female mice fed a standard or a cafeteria diet. Frozen sections from the livers of MC—male fed a standard control diet (**A**), MCaf—male fed a cafeteria diet (**B**), FC—female fed a standard control diet (**C**) and FCaf—female fed a cafeteria diet (**D**) stained with Sudan III to reveal lipid droplets (in orange) disposed along the hepatic tissue counterstained with hematoxylin-eosin (nuclei in blue). In the center of the image are centrilobular veins. Scale bar represents 50 µm.

Also reproducing the previous study [1], the Sudan III staining images of Figure 2 show that female mice had a higher number of lipid inclusions in both standard- and cafeteria diet-fed animals compared to their respective counterparts, but the increase was significantly higher under cafeteria-diet feeding. The percentage area occupied by lipids increased from 6.43 ± 0.59 in MC (Figure 2A) to 10.45 ± 0.22 in MCaf (Figure 2B) and from 9.21 ± 0.89 in FC (Figure 2C) to 18.81 ± 0.81 in FCaf (Figure 2D). FC had a 43% higher lipid area than MC, and FCaf had an 80% higher lipid area than MCaf, both of which were significant.

### 3.2. Parameters of Oxidative Stress and Antioxidant Enzyme Activities in Liver-Isolated Mitochondria

**Figure 3:**
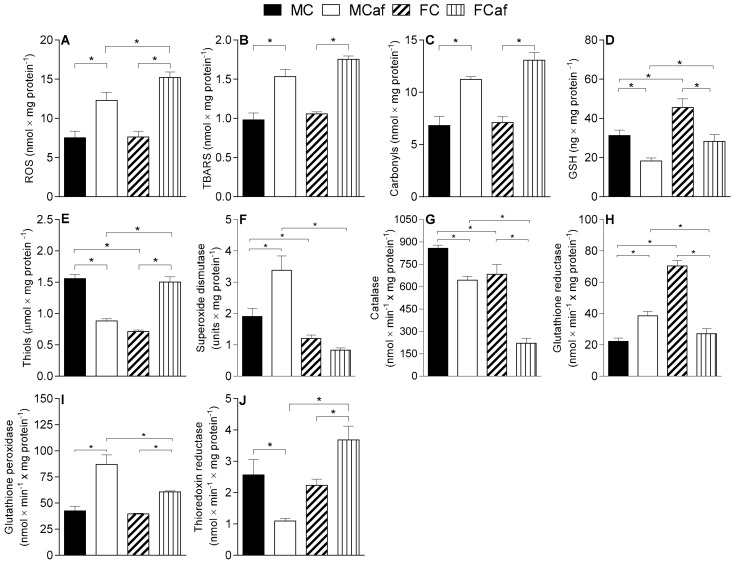
Parameters of non-enzymatic oxidative stress (**A**–**E**) and enzyme activities (**F**–**J**) of male and female mice fed a standard or a cafeteria diet. Reactive oxygen species (ROS) (**A**), lipoperoxides (**B**), protein carbonyl (**C**), reduced glutathione (GSH) (**D**) and protein thiols contents (**E**). Superoxide dismutase (**F**), catalase (**G**), glutathione reductase (**H**), glutathione peroxidase (**I**), thioredoxin reductase (**J**). Parameters were evaluated in disrupted liver mitochondria, and each value is mean ± SEM of 6–12 independent animals. * Indicates *p* < 0.05 according to ANOVA.

Mitochondria isolated from the livers of male and female mice fed a cafeteria diet (MCaf and FCaf) exhibited increased levels of total ROS when compared with their counterparts fed a standard diet (MC and FC) (Figure 3A). However, the relative increase was greater in females (+99.6%) than in males (+63.4%) compared to the FC and MC, respectively. Therefore, the amount of total ROS found in the mitochondria of FCaf was 23.7% higher than in MCaf.

The changes in mitochondrial levels of MDA and carbonylated proteins caused by the cafeteria diet were similar in both sexes. MDA levels increased by 56% in MCaf and 45.5% in FCaf when compared to their respective counterparts fed a standard diet (MC and FC, respectively) (Figure 3B). The carbonylated protein content increased by 64.7% in MCaf compared to MC and 97% in FCaf compared to FC (Figure 3C). No significant differences between the sexes were found in mitochondria from mice fed a standard or cafeteria diet.

An opposite change was induced by the cafeteria diet in the mitochondrial GSH content, with a significant decrease for MCaf (−41.5%) and FCaf (−38%) compared to the controls MC and FC, respectively (Figure 3D). Regarding the difference between sexes, females exhibited higher absolute values of GSH levels compared to both MC (+45.9%) and MCaf (+54.8%). 

In contrast with the GSH content, the content of reduced protein thiols was lower in mitochondria from females fed a standard diet (FC) in comparison with their counterpart MC (Figure 3E). In addition, the cafeteria diet induced a distinct alteration in males and females. MCaf showed a decrease of 43.4% compared to MC, while an increase of 52.5% was found in FCaf compared to FC—this showed that mitochondria from FCaf exhibited a 70.6% increase in reduced protein thiols compared to MCaf.

The activities of the antioxidant enzymes SOD, GR, GPx and CAT were measured in liver-disrupted mitochondria. SOD activity was higher in liver mitochondria from MC in comparison with FC (+58.5%), while the SOD activity increased significantly in males (+77.1%) under cafeteria diet feeding, with no change reported in females (Figure 3F). This led to an increase in activity of SOD in mitochondria from MCaf of 306.2% relative to that in mitochondria from FCaf. 

As shown for SOD activity, CAT activity was higher in MC compared to FC (+20.3%), but the change induced by the cafeteria diet was different to that seen for SOD activity. CAT activity was reduced in both FCaf (−67.6%) and MCaf (−25%) when compared to their respective counterparts fed a standard diet (Figure 3G), indicating that mitochondria from FCaf had a 65.7% lower CAT activity than those of MCaf. 

Differences between sexes and diets were found in the activity of GR. Under the standard diet, GR activity was substantially higher in FC than MC (+217%) (Figure 3H), while the opposite was found under cafeteria diet feeding. While a significant increase in the activity of GR was found in mitochondria from MCaf (+73.3%) compared to MC, the GR activity of FCaf was 62.5% lower than the standard diet-fed group (FC). These changes led to a 42% decrease in GR activity values for FCaf relative to those of MCaf. 

There was no difference between the sexes regarding GPx activity in mice fed a standard diet (MC and FC). The cafeteria diet increased the activity of this enzyme in both sexes. The increase was higher in males (+103.9%) than in females (+52.9%) compared to the respective controls fed a standard diet (Figure 3I); therefore, the GPx activity in MCaf was 43.3% higher than in FCaf. 

The activity of TrxR (Figure 3J) was also similar in liver mitochondria from males and females fed a standard diet. However, the opposite was induced by the cafeteria diet. While there was a significant decreased in the TrxR activity (−135%) in MCaf compared to MC, FCaf showed a 65% higher TrxR activity than FC. Also, TrxR activity was higher (+236.6%) in FCaf compared to MCaf.

### 3.3. Parameters of Energy Transduction in Isolated Mitochondria

To evaluate whether cafeteria diet feeding may disturb mitochondrial energy metabolism, several parameters of energy transduction were evaluated in the liver mitochondria. The effects of cafeteria diet feeding on respiration driven by succinate (FAD-dependent) and β-hydroxybutyrate (NAD^+^-dependent) were measured under two conditions: (a) just after the addition of ADP (state III respiration) and (b) after the cessation of ADP stimulation (state IV respiration); the results are shown in Table 1. None of the parameters of mitochondrial energy transduction were different in mitochondria isolated from males and females fed a standard diet when succinate was the substrate (Table 1). Significant changes were induced, however, by the cafeteria diet in both sexes. There was a decrease in the state III respiration in males (−50.2%) and females (−34.4%), as well as in state IV respiration, which reduced by 42.7% in MCaf and 24.4% in FCaf. The comparison between sexes revealed that mitochondria from FCaf exhibited higher state III (+30.0%) and state IV (+33.0%) respiration compared to those of MCaf. There was no change in the RC and ADP/O ratio. 

When β-hydroxybutyrate was the substrate, increased state III (+133.0%) and IV (110.5%) respiration was observed in females fed a standard diet compared to their respective male counterparts. In contrast to respiration driven by succinate, the cafeteria diet did not modify the state III respiration in males (MCaf) or females (FCaf), and the state IV respiration was only reduced in FCaf compared with their counterparts fed the standard diet. Regarding sex differences, state IV respiration was higher in FC (+110.5%) and FCaf (+107.1%) than in MC and MCaf, respectively. The respiratory control ratio (RC) was increased as a result of cafeteria diet feeding in MCaf, reaching values that were 45.5% and 32.4% higher than for MC and FCaf, respectively. There was no change in the ADP/O ratio.

**Figure 4:**
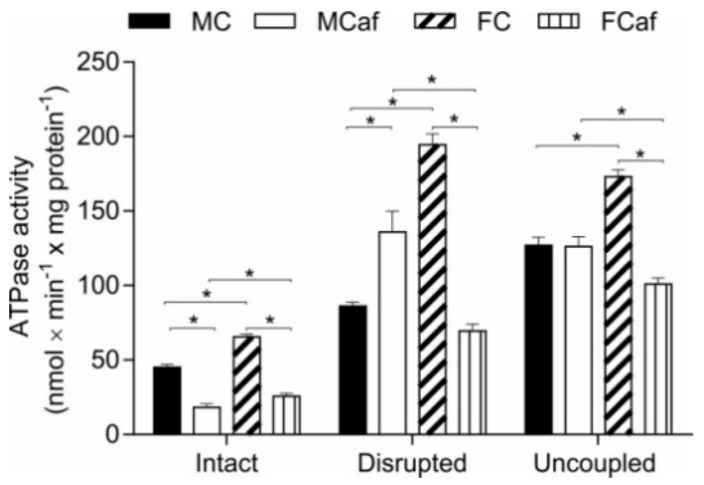
ATPase activity in liver mitochondria of male and female mice fed standard or a cafeteria diet, determined in intact coupled, disrupted and uncoupled liver mitochondria. The mitochondria were incubated at 37 °C in reaction medium as described in the Materials and Methods section. Each value represents the mean ± SEM of 4–6 independent animals. * Indicates *p* < 0.05 according to ANOVA. MC—male fed a standard control diet; MCaf—male fed a cafeteria diet; FC—female fed a standard control diet; FCaf—female fed a cafeteria diet.

The ATPase activity of isolated mitochondria was determined in three different mitochondrial preparations: intact-coupled, intact-uncoupled, and freeze-thaw-disrupted mitochondria. As shown in Figure 4, significant differences between the sexes were found in the groups for intact-coupled ATPase, since the females from the standard diet-fed group (FC) and the cafeteria diet-fed group (FCaf) had higher ATPase activity than MC and MCaf, respectively (+45% and +41%, respectively). The cafeteria diet caused a decrease in the ATPase activity of intact mitochondria of both MCaf (−59.6%) and FCaf (−60.7%), compared to their respective counterparts fed a standard diet. 

The ATPase activity of disrupted mitochondria was also significantly higher in females than in males fed a standard diet. The cafeteria diet exerted opposite effects depending on the sex of the animals. Whereas the enzyme activity was up-regulated by 57.7% in MCaf, a 64.3% inhibition was found in FCaf; these results were relative to their respective counterparts, MC and FC, respectively. Sex differences also appeared between MC and FC, with females presenting 126% higher ATPase activity, whereas obese males exhibited 95.7% higher activity than FCaf. The ATPase activity of uncoupled mitochondria was also higher in FC when compared to MC (+36.1%); the cafeteria diet only modified this enzyme activity in females (FCaf). A 41.6% reduction in ATPase activity was found in FCaf compared to FC, while MCaf exhibited a 95.7% increase in enzyme activity compared to FCaf.

### 3.4. Fatty Acid Oxidation in Isolated Mitochondria and Peroxisomes

**Figure 5:**
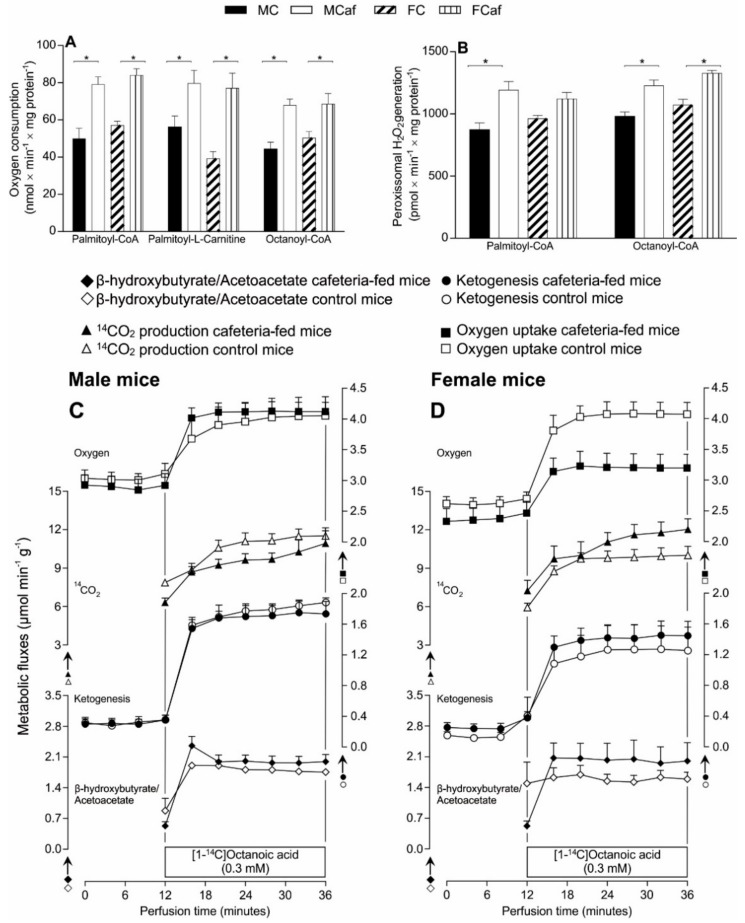
Mitochondrial (**A**) β-oxidation and (**B**) peroxisomal β-oxidation and (**C**,**D**) time courses of metabolic changes in perfused livers of male and female mice fed standard or a cafeteria diet. Liver mitochondrial fatty acid β-oxidation (**A**) was measured by polarography in the presence of 100 mM 2,4-DNP. Reactions were initiated by the addition of 20 mM octanoyl-CoA + 2.0 mM l-carnitine, 20 mM palmitoyl-CoA + 2.0 mM l-carnitine, or 20 mM palmitoyl-l-carnitine. The peroxisomal fatty acyl-CoA oxidase activity (**B**) was measured by fluorimetry (excitation at 503 nm and emission at 529 nm). This assay was based on an exogenous peroxidase-catalyzed reaction between DCFH-DA and H_2_O_2_, which resulted in the formation of a fluorescent compound. The reactions were initiated by the addition of 30 μM octanoyl-CoA or 30 μM palmitoyl-CoA. Each value represents the mean ± SEM of 4–6 independent animals. Time courses of the changes in ketone bodies and CO_2_ production and oxygen consumption in the absence and presence of exogenously added [1-^14^C] octanoic acid in livers of male (**C**) and female (**D**) mice fed standard (empty symbols) or a cafeteria diet (filled symbols). Livers of overnight fasted mice were perfused as described in Materials and methods section. The exogenous substrate was infused at the time intervals indicated by horizontal bars. Each experimental point is the mean ± SEM of 5–6 independent animals. * Indicates *p* < 0.05 according to ANOVA.

The oxidation of free fatty acids by both mitochondria and peroxisomes occurs by pathways which can generate ROS [48], particularly under the excessive supply of fatty acids from the diet. To examine the influence of cafeteria diet on the liver mitochondrial β-oxidative capacity, FA oxidation-driven oxygen consumption was measured in uncoupled mitochondria using FAs as acyl-CoA derivatives (octanoyl-CoA and palmitoyl-CoA) in the presence of carnitine and the palmitoyl-l-carnitine derivative. As shown by Figure 5A, mitochondria from males and females fed a standard diet exhibited similar rates of FA-driven respiration, irrespective of the substrates, while the cafeteria diet induced a significant increase in both males and females when compared to their respective counterparts fed a standard diet (males, palmitoyl-CoA +58.5%, palmitoyl-l-carnitine +41.8% and octanoyl-CoA +55.3%; females, palmitoyl-CoA +47.3%, palmitoyl-l-carnitine +96.5% and octanoyl-CoA +36.4%). 

The rate of peroxisomal oxidation of palmitoyl-CoA and octanoyl-CoA was evaluated through the measurements of H_2_O_2_ produced in the first step of peroxisomal oxidation (Figure 5B). The rate of H_2_O_2_production for both of the assayed fatty acids in peroxisomes from males fed a standard diet (MC) was similar to that of females (FC). For those fed the cafeteria diet, peroxisomes from MCaf oxidising palmitoyl-CoA exhibited higher values (+36.7%) compared to MC, without a significant change in FCaf. When the substrate was octanoyl-CoA, the peroxisomal oxidation was increased by 25.2% and 23.7% in MCaf and FCaf, respectively, compared to the standard diet-fed counterparts.

### 3.5. Oxygen Consumption, ^14^CO_2_ Production and Ketogenesis in Intact Livers

The steady-state fluxes of exogenous fatty acid oxidation can be measured in intact perfused livers. In the experimental series, a perfusion fluid containing octanoic acid plus traces of octanoate labelled with the ^14^C isotope was infused in livers from fasted mice from the four groups (MC, FC, MCaf and FCaf); effluent perfusates were collected for the measurement of ketone bodies and CO_2_ production, as well as oxygen consumption. The release of ^14^CO_2_ in the effluent liquid reflects the metabolic fluxes through the fatty acid β-oxidation pathway and citric acid cycle; the production of acetoacetate and β-hydroxybutyrate indicates the ketogenic activity, while the β-hydroxybutyrate/acetoacetate ratio indicates the mitochondrial redox state [49]. Moreover, the increase in oxygen consumption due to octanoate influx is a result of metabolic fluxes from the oxidation of exogenous octanoate to the mitochondrial respiratory chain. 

The infusion of exogenous octanoate (0.3 mM) plus traces of [1-^14^C]octanoate in perfused mice livers caused an increase in ketone bodies, CO_2_ production and oxygen consumption in the four groups of mice (Figure 5, panels C–D). Panel C of Figure 5 shows that no significant differences were found in the steady-state fluxes of all metabolic parameters (before and after octanoate infusion) when comparing the males fed a standard (MC) diet to males fed a cafeteria diet (MCaf). In females (panel D), however, significant differences were observed in the steady-state rates of oxygen consumption. A reduced rate (−27.4%) was found in females fed a cafeteria diet (FCaf), with no changes in ketogenesis and CO_2_ production when compared to their counterparts fed a standard diet (FC). The rate of oxygen consumption at the terminus of octanoate infusion in livers from FC was 4.074 µmol × min^−1^ × g^−1^, while in FCaf it was 3.197 µmol × min^−1^ × g^−1^ (*p* = 0.0181).

In the comparison between sexes (panel A versus panel B), the only significant difference was in the steady state rates of oxygen consumption in mice fed a cafeteria diet, where FCaf had lower rates (−29%) than MCaf. At the terminus of octanoate infusion, the steady state flux in livers from FCaf was 3.197 µmol × min^−1^ × g^−1^ compared to 4.122 µmol × min^−1^ × g^−1^ in MCaf (*p* = 0.01).

## 4. Discussion

The present study provided evidence that mitochondria contribute to sex differences in liver steatosis and oxidative stress in mice fed a cafeteria diet. Confirming our previous observations [1], female mice fed a cafeteria diet (FCaf) displayed more pronounced hepatic steatosis compared to their male counterparts (MCaf), despite having a similar increase in body weight gain and relative increase in adiposity index. Mitochondria may be implicated in liver oxidative stress, which has been reported to be higher in FCaf than in MCaf [1], as indicated by the increased ROS content compared to that of MCaf. Evidence of ROS-induced oxidative damage was also found, as the content of TBARS was increased in mitochondria from both FCaf and MCaf. TBARS, including malonaldehyde (MDA) and 4-hydroxynonenal (4-HNE), are highly reactive molecules which can oxidise other cellular components [50]. Therefore, these molecules are considered second messengers of oxidative stress due to their capacity to diffuse from their site of formation [51,52] and amplify oxidative stress. As a consequence of the attack by ROS or by lipid peroxidation products, there is irreversible damage to proteins through the non-enzymatic addition of carbonyl groups [50,53]; this was also demonstrated in the current study to be higher in the mitochondria from FCaf and MCaf. 

It seems, therefore, that mitochondria contribute to the increased content of TBARS found in whole liver homogenates of FCaf, as previously reported [1]. However, we found no significant differences between the sexes in the mitochondrial content of TBARS and carbonyl proteins (Figure 3B,C); therefore, the greater oxidative damage found in FCaf liver than in MCaf was not reproduced in isolated mitochondria. There are probably other ROS-generating sites which contribute to oxidative damage to a greater extent in the whole livers of FCaf [1].

Although mitochondria are the site of the majority of cellular ROS generation [54], the ER also generates ROS [8]. In mitochondria, ROS are produced as a by-product of oxidative metabolism linked to ATP generation, whereas the production of ROS in the ER mainly happens during protein folding, as a result of disulphide bond formation [55]. The fact that the ER may actually contribute to excess ROS generation in the livers of FCaf is supported by our previous observation that FCaf livers, but not MCaf livers, show increased mRNA expression of nuclear factor erythroid 2–related factor 2 (*Nrf2*), a transcription factor that is responsible for antioxidant cell response, and heme oxygenase 1 (*Hmox1*), a gene containing the antioxidant response element (ARE) [1]. Another organelle which can contribute to ROS production in liver cells is the peroxisome [56]. It is unlikely, however, that peroxisomes are implicated in the exacerbated oxidative stress of FCaf livers, even though we have observed a higher peroxisomal rate of H_2_O_2_ generation from fatty acid β-oxidation in MCaf and FCaf (Figure 5B), with no differences observed between the sexes. 

Sex differences in the mitochondrial antioxidant capacity also contribute to the increased sensitivity of FCaf livers to cellular oxidative stress. A more effective neutralisation of ROS was found in mitochondria from males fed a cafeteria diet (MCaf), as indicated by the increased activities of SOD (Figure 3F), GR (Figure 3H) and GPx (Figure 3I) compared to males fed a standard diet (MC). Mitochondrial SOD catalyses the dismutation of superoxide (O_2_^−^) into hydrogen peroxide (H_2_O_2_), thereby maintaining a low concentration of (O_2_^−^), while CAT and GPx remove the H_2_O_2_ generated from the SOD reaction [57]. Mitochondrial GPx catalyses both the reductive detoxification of peroxides and, in some situations, the catalytic transfer of oxidation equivalents to another protein. GPx requires reduced GSH for activity [58] and GR reduces the oxidised GSSG using the reductive force of NADPH to restore GSH levels [55,59].

In FCaf mitochondria, besides the absence of SOD activation, the GR activity was reduced compared to that in their male counterparts (Figure 3H). Additionally, catalase activity was found to be reduced in FCaf to a higher degree compared to the decrease observed in MCaf (Figure 3G). The increase in GPx activity, which also neutralises the H_2_O_2_ produced in mitochondria, possibly compensates for the reduced activity of CAT in both MCaf and FCaf; it should be noted that this enzyme was activated to a lesser extent in FCaf than in MCaf animals. 

It should be mentioned that our previous study evaluated the SOD, CAT and GPx activities in liver homogenates, which assesses the enzyme activities irrespective of their compartmentalisation [1]. In liver homogenates, there was (a) no change in SOD activity in either males or females; (b) a relative decrease in CAT activity due to the cafeteria diet, similar to the findings of this study; and (c) increased GPx activity in FC compared to MC, while the cafeteria diet induces a relative reduction in its activity in both FCaf and MCaf [1]. In the current study, however, the GPx activity in mitochondria isolated from FC and MC was similar and the cafeteria diet induced an increase in activity in both males and females, which was greater in MCaf. 

GPx1, 3 and 4 are located in mitochondria and are components of the NADPH-dependent GSH-based antioxidant system. GPx7 and 8 in the ER play a different role [60]. The enzymes participate in protein folding-associated disulphide bond formation [61] during protein folding in the ER in eukaryotes. After protein thiol oxidation by protein disulphide isomerase (PDI), PDI can be oxidised by several processes, including those which are peroxidase-driven (such as novel GPx7/8). In this reaction, GPx enzymes utilize the H_2_O_2_ generated by ER oxidase-1 (Ero), which uses molecular oxygen as the electron acceptor in another pathway of PDI oxidation [55]. It seems that there is a predominance of ER GPx activity in the whole liver of mice fed a cafeteria diet, which would account for the reduction in activity. This results in H_2_O_2_ neutralisation impairment and an imbalance in the ER redox state in FCaf livers.

A reduction in mitochondrial GSH content was seen in both MCaf and FCaf (Figure 3D). Females fed a standard diet (FC) had higher levels of mitochondrial GSH than MC, a finding which correlated with the higher GR activity in FC. Under cafeteria diet feeding, the reduction in mitochondrial GSH content in males (MCaf) was associated with higher GPx and GR activities, suggesting a greater demand for neutralising ROS-induced oxidative damage when compared to MC. In FCaf, however, the reduced level of GSH was associated with a significant reduction in GR activity and a lower activation of GPx activity. These findings suggested that, besides the increased generation of ROS, mitochondria from FCaf showed an impaired capacity for peroxide detoxification and the restoration of the GSH content compared to MCaf mitochondria. 

GSH is the most abundant thiol in the liver and one of the major electron donors for disulphide reduction reactions. Other small proteins can also provide reduced power to cellular reduction reactions, including members of the thioredoxin (Trx) and glutaredoxin (Grx) families [60]. Trx proteins also have active-site dithiols that donate reducing power to select substrates, thereby becoming disulphides. Trxs can be reduced back to dithiols by thioredoxin reductases (TrxR), which are also NADPH-dependent. The measurement of TrxR activity in mitochondria revealed a difference between the sexes in response to cafeteria diet feeding (reduced activity in MCaf and up-regulation in FCaf) when compared to their respective counterparts fed a standard diet (Figure 3J). These differences were in accordance with the content of reduced thiol proteins, which was reduced in MCaf and increased in FCaf (Figure 3E). An association between the increased generation of ROS and the reduction of thiol content has been reported in other models of diet-induced obesity [62,63]. A possible explanation for the effect observed in FCaf is that the thioredoxin (Trx)-based antioxidant system was activated as a way of compensating for a decrease in the GSH-based antioxidant system in FCaf, a condition that has been described in tumour cells with oxidative stress alterations [64].

Taken together, these data provide evidence that mitochondria are involved in the increased cellular oxidative stress induced by the cafeteria diet in females, a condition which we have demonstrated, in our previous study, to be a consequence of unresolved ER stress [1]. This finding is in accordance with the suggestion that an excess of mitochondrial ROS generation can induce ER stress, while ER stress can generate oxidative stress and affect mitochondria in a reciprocal manner [65,66]. This vicious cycle may aggravate oxidative damage and metabolic disorders, such as those found in FCaf livers, which also show some evidence of hepatocyte ballooning and inflammation as previously demonstrated via histological analysis [1].

In order to investigate whether these sex differences are related to an impairment in the efficiency of mitochondrial energy transduction, several parameters were measured in mitochondria isolated from both female and male mice fed standard or cafeteria diets. Isolated mitochondria from FCaf and MCaf livers exhibited a lower state III respiration rate driven by succinate when compared with their control counterparts, with no differences between the sexes. However, when β-hydroxybutyrate was used as a substrate, state III respiration was substantially higher in FC compared to MC; the cafeteria diet did not provoke relative changes in the respiratory rates of either sex. It should be mentioned that differences in mitochondrial bioenergetic parameters are not uncommon when mitochondria are oxidising complex I or II substrates (Table 1). These variations are related to differences in the stoichiometry of protons translocated per electron and the basal proton leak through membranes, which influence the rate of respiration and the proton motive force (Δp) [67].

Despite these sex and diet differences, there were no significant alterations in the respiratory control coefficient (RC) and ADP/O ratios between the four groups of mice (Table 1), indicating the absence of a disturbance in the coupling between the electron respiratory chain and ADP phosphorylation. 

Nevertheless, the reduced rates of succinate-driven respiration observed in mitochondria from both FCaf and MCaf could have contributed to a high content of mitochondrial ROS in these mitochondria compared to those of their counterparts fed a standard diet. Most of the cellular ROS is generated by the mitochondria, and several sites of superoxide and/or hydrogen peroxide have been identified in mammalian mitochondria [68]. A reduction in respiration is associated with a high proton electrochemical gradient (Δp) and alterations in the redox state of components of the respiratory chain, particularly coenzyme Q, which favours superoxide formation. Interestingly, mitochondrial ROS production is more sensitive to the electrical components of Δp when the substrate is succinate but not when complex I substrates are used [69], supporting the assumption that the reduced respiration driven by succinate may have contributed, in part at least, to the increased ROS content in the mitochondria of mice fed a cafeteria diet. 

Similar to state III respiration, the ATPase activity in intact coupled mitochondria is under the control of the Δp driven by the reverse of the FoF1-ATP synthase complex [67]. Under this condition, a reduction in ATPase activity was found in the mitochondria from both FCaf and MCaf (Figure 4). In uncoupled mitochondria and in freeze-thawing disrupted mitochondria, the ATPase activity is no longer controlled by Δp and, thus, the maximal capacity of ATPase can be achieved. In uncoupled mitochondria, in contrast to disrupted mitochondria, the adenine nucleotide translocase is a necessary step for enzyme activity. Differences between the sexes were found regarding the maximal activity of ATPase. Whereas a significant decrease in the maximal capacity of ATPase was found in mitochondria from FCaf livers, as evidenced by the decreased activity in both uncoupled and disrupted mitochondria, the maximal activity of ATPase in mitochondria from MCaf livers was stimulated, with no changes in enzyme activity in uncoupled mitochondria.

The decreased activity of ATPase in mitochondria under controlled conditions could represent the decreased efficiency of the FoF1-ATP synthase complex for catalysing oxidative phosphorylation. An expected response to this effect is a reduction in state III respiration relative to state IV respiration, i.e., a decrease in the RC coefficient. However, such an alteration was not found in mitochondria from cafeteria diet-fed mice, irrespective of the substrate oxidised. There are no simple explanations for these discrepancies, but a reduction in the maximal catalytic activity of ATPase in the mitochondria from FCaf may have negative consequences for the livers under specific situations of high energy requirements. 

The measurements of fatty acid oxidation in the isolated organelles, mitochondria and peroxisomes, along with metabolic fluxes due to octanoate oxidation in the perfused intact livers, further supported the finding that the cafeteria diet did not impair the efficiency of mitochondria in liver energy metabolism. The oxidation of palmitoyl-CoA, palmitoyl-l-carnitine and octanoyl-CoA was measured in uncoupled mitochondria and the oxygen consumption rates were higher in cafeteria diet-fed mice, with no differences between the sexes. In these assays, the rates of oxygen consumption reflect the maximal capacity of fatty acid oxidation, as the mitochondrial respiratory chain was not controlled by the proton electrochemical gradient; therefore, there was no restriction for the oxidation of FADH_2_ and NADH generated in the β-oxidative pathways and in the oxidation of acetyl-CoA through the citric acid cycle [70]. The increased rates of oxygen consumption driven by these three different fatty acids clearly demonstrated that, under cafeteria diet feeding, fatty acid β-oxidation was increased without limitation by the citric acid cycle or the respiratory chain. The peroxisomal β-oxidation of palmitoyl-CoA and octanoyl-CoA was also stimulated by the cafeteria diet and the stimulus was similar in both FCaf and MCaf, as observed for mitochondrial fatty acid oxidation.

In our previous study, we found increased mRNA expression of *Cpt1a* and *Acox1* in FCaf livers [1], an observation which is in agreement with the increased mitochondrial and peroxisomal fatty acid oxidation in FCaf livers compared with their control counterparts. Since these changes in mRNA expression were not observed in MCaf livers [1], it can be suggested that there are additional regulatory mechanisms influencing the increase in mitochondrial and peroxisomal fatty acid oxidation under cafeteria diet feeding. 

Increased fatty acid oxidation has also been reported in several rodent models and in human subjects with obesity [71,72,73]. It has been proposed that the increased absorption of free fatty acids, whether from diet or hepatic synthesis through an excess of carbohydrates in the diet, can be balanced by an increased rate of mitochondrial β-oxidation [74] in order to protect hepatocytes from lipotoxicity due to excessive fatty acid deposition [75]. 

Our data did not agree with this proposition, since FCaf livers had higher TG contents than MCaf livers, despite the similar rates of mitochondrial and peroxisomal fatty acid oxidation (Figure 5A,B). Instead, the absence of this regulatory mechanism corroborates our previous hypothesis that the greater steatosis in FCaf livers is not directly linked to changes in enzymes responsible for fatty acid or triglyceride metabolism, but is a consequence of an impairment of the cafeteria diet-induced UPR pathway, which induced several responses including the lower release of VLDL from livers [1].

In the intact liver, under perfusion, no significant differences between sex and diet were found with regard to fatty acid oxidation (Figure 5C,D). The steady state rates of ^14^CO_2_ and ketone body production from exogenous octanoate were similar between the four groups of mice. This indicates that the acetyl-CoA generated in the β-oxidation of octanoate was subsequently oxidised in the tricarboxylic acid cycle, and also that the fraction of acetyl-CoA destined for the production of ketone bodies was not altered by the cafeteria diet in both males and females. Nevertheless, a significant difference was found in the rate of oxygen consumption, as livers from FCaf exhibited a decrease in the rate of oxygen consumption due to octanoate infusion when compared with the livers of FC, an effect that was not observed in males. This reduction in the steady state rate of oxygen consumption from FCaf was not accompanied by a reduction in ^14^CO_2_ production or in the production of ketone bodies, suggesting an increase in mitochondrial Δp, a condition which has been demonstrated to favour mitochondrial ROS generation [69]. 

Taken together, the examination of fatty acid oxidation in isolated organelles and in perfused mice livers suggested that the cafeteria diet increases the maximal enzymatic capacity of organelles to oxidise fatty acids; however, in the intact liver, under steady-state conditions, such a stimulus seems not to be physiologically relevant. It should be mentioned, however, that the perfusion experiments were performed in livers from fasted mice. It is expected that, during cafeteria diet feeding, the livers receive a high amount of fatty acids from the diet; therefore, an increase in the maximal capacity of organelles could be part of a regulatory mechanism which allows the rapid adjustment to excess fatty acids in the diet. 

As a result, we have not found evidence that the cafeteria diet impairs mitochondrial energy metabolism, even in FCaf livers which have higher steatosis and cellular oxidative stress than MCaf livers. Therefore, a link between ER stress and mitochondrial energy alterations does not seem to occur in cafeteria diet-induced ER stress in FCaf, as has been suggested in studies in which ER stress or mitochondrial energy disruption are induced by drugs in isolated cells [14,15]. It is also unlikely in our experimental scenario that a mitochondrial calcium overload due to interaction with the ER has caused hepatocyte death [12,13,15,76]. Besides a lack of perturbation in the mitochondrial energy metabolism, we did not find in our previous study any evidence of fibrosis or apoptotic cells in the liver parenchyma of FCaf, or changes in the plasma levels of AST and ALT [1]. 

It can be concluded that the increase in mitochondrial ROS generation associated with a decrease in the antioxidant defence capacity may have led to an excess of ROS-derived toxic metabolites in the whole cells of FCaf livers, which probably induced or reinforced the ER stress which was previously characterised in FCaf livers [1]. In males, the reduced mitochondrial ROS generation and the effective mitochondrial antioxidant enzyme system may be part of the mechanism linked to the branch of UPR involving Fgf21 up-regulation, which we have previously suggested to be effective for resolving the ER stress only in males fed a cafeteria diet. Although playing a role in the establishment of cellular redox imbalance in FCaf livers, mitochondria preserved their role in liver energy metabolism.

## Figures and Tables

**Table 1:** The influence of cafeteria diet-induced obesity on mitochondrial oxygen consumption.

Substrate	Parameter	Male Mice		Female Mice	
		*Control*	*Cafeteria*	*Control*	*Cafeteria*
**Succinate**	**state III**	261.11 ± 7.50	129.86 ± 5.65 ^D^	256.92 ± 7.36	168.56 ± 8.77 ^DS^
	**state IV**	55.12 ± 3.39	31.60 ± 2.25 ^D^	55.61 ± 3.13	42.02 ± 2.17 ^DS^
	**RC**	4.66 ± 0.40	5.23 ± 0.52	3.43 ± 0.34	3.93 ± 0.25
	**ADP/O**	2.14 ± 0.18	2.15 ± 0.22	2.19 ± 0.23	2.05 ± 0.24
**β-Hydroxybutyrate**	**state III**	69.29 ± 2.83	73.54 ± 4.52	162.02 ± 4.64 ^S^	172.78 ± 11.46 ^S^
	**state IV**	25.51 ± 2.65	21.73 ± 2.85	53.71 ± 1.52 ^S^	45.0 ± 3.16 ^DS^
	**RC**	3.52 ± 0.16	5.12 ± 0.39 ^D^	3.06 ± 0.18	3.46 ± 0.17 ^S^
	**ADP/O**	2.72 ± 0.20	2.72 ± 0.13	3.33 ± 0.29	3.50 ± 0.38

Data are the mean ± standard errors of 6–7 experiments with identical protocol. The rate of oxygen consumption in the state III and state IV respiration driven by β-hydroxybutyrate and succinate in the presence and absence of exogenously added ADP were expressed as nmol × min^−1^ × mg protein^−1^. The respiratory control ratio (RC) and the ADP/O ratio were calculated according to the method described by [35]. ANOVA (*p* < 0.05): ^S^ and ^D^ indicate effect of sex and effect of cafeteria diet, respectively.
